# Thermophysical Properties’ Enhancement of LiNO_3_-NaNO_3_-KNO_3_-NaNO_2_-KNO_2_ Mixed with SiO_2_/MgO Nanoparticles

**DOI:** 10.3390/ma17184611

**Published:** 2024-09-20

**Authors:** Chuang Zhu, Minhao Xu, Baiyuan Tian, Manting Gu, Li Gong

**Affiliations:** 1 School of Energy and Electrical Engineering, Qinghai University, Xining 810016, China; 18283138903@163.com (M.X.); 17697275434@163.com (B.T.); gumanting2023@163.com (M.G.); gongli@qhu.edu.cn (L.G.); 2 Engineer School, Qinghai Institute of Technology, Xining 810016, China

**Keywords:** molten salt, nanoparticle, composite material, specific heat, thermal conductivity

## Abstract

The aim of this study is to further enhance the thermal storage and heat transfer performances of a low-melting-point quinary salt. The eutectic salt was prepared using LiNO_3_, NaNO_3_, KNO_3_, NaNO_2_, and KNO_2_ as raw materials, followed by the doping of nano-SiO_2_ and nano-MgO into the base salt using a microwave-assisted method. The thermal properties of the samples were analyzed using a Synchronous Thermal Analyzer and a Laser Flash Apparatus. The co-doping of two types of nanoparticles was found to significantly enhance the specific heat capacity of the base salt. The maximum specific heat reached 2.36 J/(g·K), showing a 50.4% increase compared to the base salt. The thermal conductivity of molten salts can be affected by nanoparticles. An observed sample demonstrated a thermal diffusivity of 0.286 mm^2^/s, indicating a 19.2% improvement over the base salt, which may be attributed to enhanced phonon thermal efficiency. In addition, this study revealed that while interfacial thermal resistance can enhance specific heat capacity, it can also lead to a decrease in the thermal conductivity efficiency of materials. This work can offer insights and references for the enhancement of molten salt properties.

## 1. Introduction

The development and utilization of clean energy is advantageous for protecting the global ecological environment and for alleviating the constraints posed by the limited reserves of fossil fuels. In the future development of human society, clean energy will be the main form of energy required. Despite being a vital part of clean energy sources with limitless potential, solar energy’s drawbacks lie in its variability and instability, posing a challenge to meeting the consistent energy demands in industrial and domestic settings. Thermal storage technology plays a vital role in tackling the challenge of solar energy utilization by storing thermal energy for subsequent release or conversion into alternative energy forms like electricity [1]. Multiple forms of energy storage exist, ranging from mature technologies like pumped hydro storage and compressed air energy storage to innovative prospects like Pumped Heat Energy Storage [2]. In recent years, the integration of various thermal energy storage technologies has shown promising application prospects. Palacios et al. [3] integrated the three thermal storage methods of sensible heat, latent heat, and thermochemical-based TES into one system, which has great potential for storing heat (up to 2 GJ∙m^−3^). Funayama et al. [4] combined a molten-salt thermocline with thermochemical energy storage, and the novel hybrid system shows superior thermal discharge performance.

Molten salt is a commonly used heat storage material in thermal energy storage technologies, known for its high heat storage density, strong thermal stability, low vapor pressure, and cost-effectiveness [5,6]. The enhancement of thermal storage and heat transfer properties in molten salts stands out as a pivotal area of research in this field, bearing essential significance in improving the efficiency of thermal utilization systems.

It has been observed in research that the incorporation of nanoparticles typically aids in boosting the specific heat of molten salt, consequently elevating the thermal energy storage density. Zhang et al. [7] added 0.5 wt.% nano Al_2_O_3_ and 0.3 wt.% nano graphite powder into solar salt, resulting in a composite material with a specific heat capacity 21.79% higher than that of the base salt, reaching 1.90 J/(g·K). Li et al. [8] dispersed 1 wt.% SiO_2_ nanoparticles into solar salt using a two-step method, and observed that the average specific heat of the mixed nanofluid was 1.84 J/(g·K), representing a 23% increase compared to the base salt. Jeong et al. [9] prepared KNO_3_-SiO_2_ nanofluids and observed an increase in the specific heat capacities of the solid and liquid phases to 1.64 J/(g·K) and 1.78 J/(g·K), respectively, representing enhancements of 24.1% and 28.1%. Dudda et al. [10] investigated the effect of incorporating 1 wt.% nanoscale particles of varying sizes into solar salts. They found that the addition of 60 nm SiO_2_ resulted in the highest increase in specific heat capacity, leading to a solid phase-specific heat of 1.34 J/(g·K) and a liquid phase-specific heat of 1.81 J/(g·K), representing enhancements of 10.7% and 23.1%, respectively.

Additionally, the addition of nanoparticles commonly results in increased heat transfer efficiency in molten salts. Meng et al. [11] investigated the impact of 20 nm SiO_2_ on solar salt, revealing that the addition of 1 wt.% SiO_2_ enhances the thermal conductivity to 0.700 W/(m·K). Yu et al. [12] blended MgCl_2_-NaCl-KCl with expanded graphite to form a composite material. They observed that the addition of 1 wt.% SiO_2_ nanoparticles enhanced the thermal conductivity of the composite material to 6.03 W/(m·K), representing a 20.7% improvement over the original composite material. Chen et al. [13] incorporated 0.5 wt.% nano SiO_2_ into Ca(NO_3_)_2_-KNO_3_-NaNO_3_-LiNO_3_ and observed a favorable thermal conductivity of the resulting material (0.528 W/(m·K)).

It should be noted that the mechanism through which nanoparticles strengthen the thermal properties of molten salts is still under research and has not been unanimously agreed upon. Abir and Shin [14] employed molecular dynamics to investigate the mechanism by which SiO_2_ enhances the specific heat of solar salt, and they found that the increase in specific heat may be attributed to the heightened surface area and surface energy of the nanofluid. Li et al. [15] suggested that nanoparticles may lead to increased constraints on ion motion, potentially contributing to the enhancement of specific heat capacity. Yang et al. [16] conducted a simulation study on the mechanism of specific heat enhancement, revealing a potential correlation between specific heat increase and the coordination number of cations and anions. Rong et al. [17] investigated the mechanism of heat transfer enhancement, suggesting that the Brownian motion of particles induces the micro-convection of inorganic salt ions around them, thereby increasing their thermal conductivity. Although the mechanism is still being studied, it is clear that nanoparticles are often beneficial in enhancing the thermal properties of molten salts. Currently, research on the changes in material properties resulting from the simultaneous doping of two types of nanoparticles is insufficient.

This study aims to explore the influence of doping two types of nanoparticles on the thermal properties of low melting point phase change materials (LiNO_3_-NaNO_3_-KNO_3_-NaNO_2_-KNO_2_). Materials with such a low melting point have the potential to be applied in everyday life. A series of composite materials consisting of inorganic salts, nano-SiO_2,_ and nano-MgO have been prepared through a microwave-assisted method. This method offers advantages such as the uniform heating of samples, ease of automated control, and rapid heating rates. The microwave facilitates improved dispersion of nanoparticles in the molten salt, thereby reducing nanoparticle agglomeration [18]. The utilization of nano-SiO_2_ and nano-MgO in this research is based on two factors: their favorable cost-effectiveness and their high frequency of use in this field, facilitating comparative analysis by other researchers. The nanoparticles were analyzed using SEM, TEM, and XRD. The thermal storage and heat transfer properties were studied using a Synchronous Thermal Analyzer and a Laser Flash Apparatus, respectively. Finally, by integrating the properties of the materials with their microstructures, the potential mechanism underlying the alteration of material performances was discussed.

## 2. Materials and Methods

### 2.1. Materials

The eutectic salt utilized in this study is LiNO_3_-NaNO_3_-KNO_3_-NaNO_2_-KNO_2_ (33.5 wt.%-1.2 wt.%-1.2 wt.%-17.4 wt.%-46.7 wt.%). The melting point of this base salt is 74.4 °C and the decomposition temperature is 508 °C [19]. These experimental materials were purchased from Aladdin Biochemical Technology Co., Ltd. (Shanghai, China). The nanoparticles used were SiO_2_ and MgO, both sourced from the China National Pharmaceutical Group Corporation Chemical Reagent Company (Shanghai, China). The parameters of the inorganic salts and nanoparticles used are detailed in Table 1 and Table 2, respectively.

The inorganic salts were uniformly mixed in the prescribed ratios, followed by heating in a muffle furnace at 200 °C for 24 h. The resulting material was then pulverized into powder, thereby producing the base salt.

**Figure 1:**
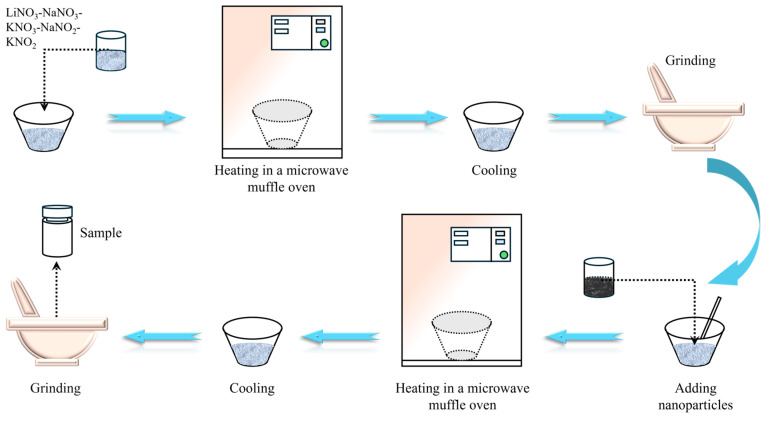
Preparation process of molten salt nanocomposites.

In this study, microwave irradiation was employed for the synthesis of molten salt nanocomposites. First, nanoparticles and base salt were proportionally weighed and homogeneously ground in a mortar. Subsequently, the mixture was heated in a microwave muffle furnace to 200 °C and kept at a constant temperature for 15 min. Finally, the material was retrieved, cooled, and ground into powder. The preparation process is depicted in Figure 1. The nanoparticle contents in different samples are presented in Table 3.

### 2.2. Measurement Apparatuses and Methods

#### 2.2.1. Testing of Heat Storage Property

The melting point, latent heat, and specific heat of samples were measured using the Synchronous Thermal Analyzer (STA-449F3, NETZSCH, Selb, BAV, Germany). The measurement conditions were as follows: the initial temperature was set at 30 °C and maintained for 15 min, followed by heating at a rate of 10 °C/min until reaching 400 °C, and then maintained for another 15 min. A helium gas atmosphere was used during the testing, with a protective gas flow rate of 20 mL/min and a purge gas flow rate of 50 mL/min. Each sample was measured three times to ensure the accuracy of the test results.

#### 2.2.2. Testing of Heat Transfer Property

The thermal diffusivity was measured using a Laser Flash Apparatus (LFA-467HT, NETZSCH). A platinum–rhodium crucible with a diameter of 12.7 mm was utilized for the tests, with argon gas employed for both purging and protection at a flow rate of 50 mL/min. The testing temperature ranged from 50 °C to 400 °C. Each sample was measured thrice to ensure the precision of the test results. The thermal conductivity can be calculated using Equation (1):

λ = ρ·cp·α,(1)

where λ represents the thermal conductivity, ρ density, cp specific heat, and α thermal diffusivity.

#### 2.2.3. Microstructure

Microstructures of the samples were observed using a field emission scanning electron microscope (JSM-7900F, JEOL Ltd., Tokyo, Japan). During sample preparation, the samples were fixed onto a sample holder using a conductive adhesive. Following this step, a 60-s gold coating was applied to the samples using an ion-sputtering coater (KYKY SBC-12, Zhongke Technology Co., Ltd., Beijing, China). Lastly, SEM images were taken in a high vacuum environment using a secondary electron detector. The morphology and dimensions of the nanoparticles were observed using scanning electron microscopy (JSM-7900F) and transmission electron microscopy (Talos F200X, Thermo Fisher Scientific, Waltham, MA, USA). Additionally, the composition and crystalline state of the nanoparticles were analyzed using an X-ray diffractometer (D/max2500PC, Rigaku Corporation, Tokyo, Japan).

## 3. Results and Discussion

### 3.1. Characteristics of Nanoparticles

**Figure 2:**
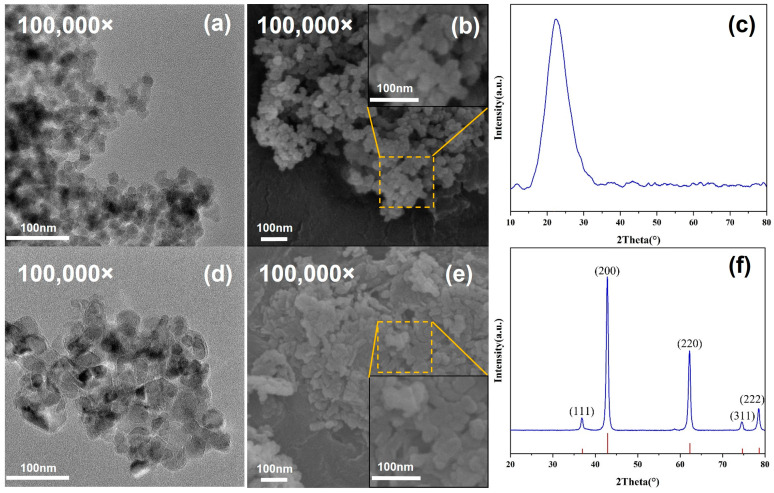
(**a**) TEM image, (**b**) SEM image, and (**c**) XRD of nano-SiO_2_; (**d**) TEM image, (**e**) SEM image and (**f**) XRD of nano-MgO.

From Figure 2, it is evident that the nano-SiO_2_ particles are spherical with a diameter of about 30 nm, exhibiting an amorphous structure. The nano-MgO nanoparticles exhibit a polyhedral or spherical shape with a particle size of about 50 nm, displaying crystalline characteristics. Strong diffraction peaks at 36.94°, 42.92°, 62.30°, 74.69°, and 78.63° are seen due to the feature peaks (111), (200), (220), (311), and (222) of MgO (JCPDS No. 45-0946), respectively.

### 3.2. Melting Point and Latent Heat of Phase Change

**Figure 3:**
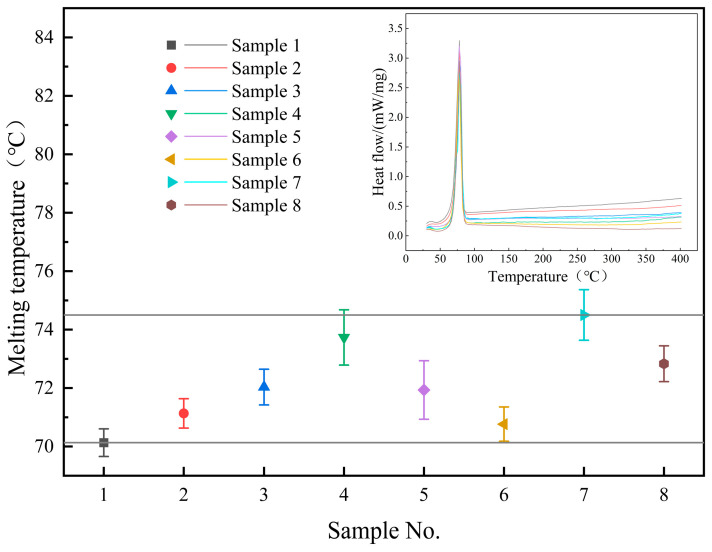
Melting temperature and DSC curve of the samples.

Figure 3 displays the melting points of the samples, revealing a consistent range of 70–75 °C for all samples, in close agreement with the literature’s value of 74.4 °C [19]. After the addition of nanoparticles, the melting point of the molten salt slightly increased, with the sample containing 0.9% SiO_2_ and 0.1% MgO nanoparticles showing the highest melting point at 74.5 °C.

**Figure 4:**
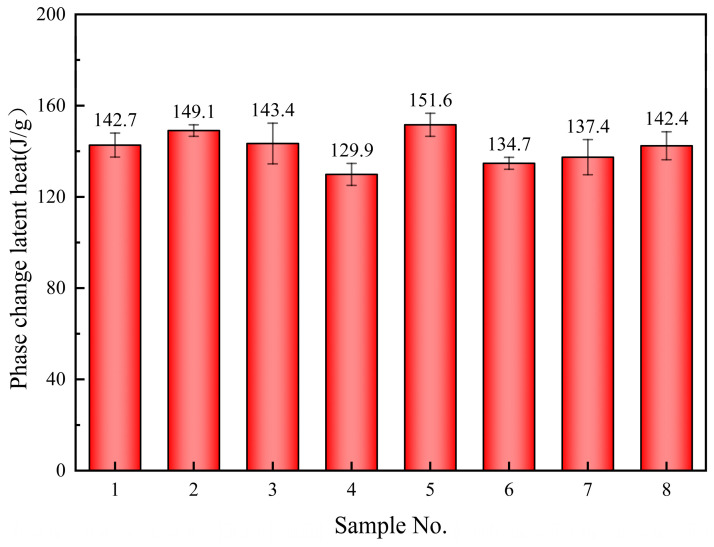
Phase change latent heat of the samples.

The phase change enthalpy of the samples is predominantly distributed between 130 and 150 J/g, as depicted in Figure 4. The addition of 0.5% SiO_2_ and 0.5% MgO nanoparticles results in the highest latent heat of the composite material, reaching 151.6 J/g, which represents a 6.2% increase compared to the base salt. It is also observed that the latent heats of samples 4, 6, 7, and 8 are lower than that of the base salt, with sample 4 exhibiting the lowest latent heat.

The melting point and latent heat of a sample are closely linked to its lattice energy, which is clearly affected by the doping of nanoparticles. This influence is derived from at least the following aspects. Firstly, the surface effect of nanoparticles induces lattice distortion, thereby influencing the inorganic salt ions near the surface. Secondly, the surface charge of nanoscale particles may induce electric field forces that impact the distribution of inorganic salt ions at a considerable distance [20]. Furthermore, nanoparticles are a component of the samples; however, unlike the salt, they do not undergo a phase change during heating, thereby impacting the latent heat value.

### 3.3. Specific Heat Capacity

**Figure 5:**
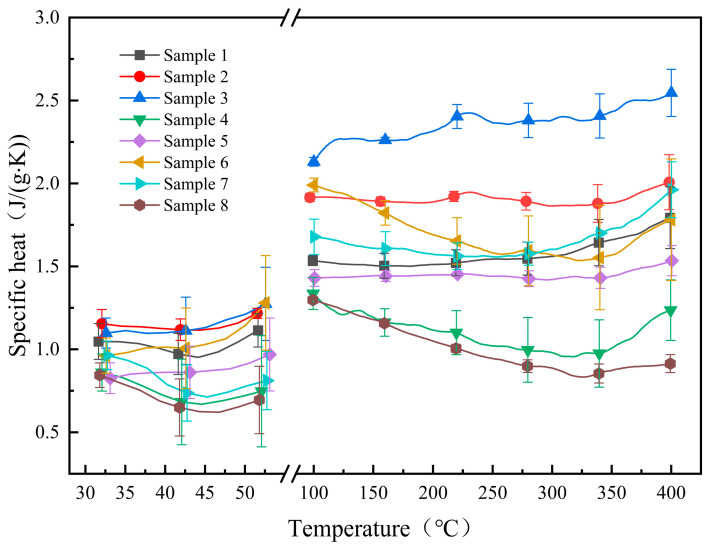
Specific heat curves of samples in solid and molten states.

The specific heat and standard deviations of all samples are listed in Table 4, and the specific heat curves of the samples in both solid and molten states are displayed in Figure 5. Compared to the base salt, samples 2 and 3 exhibit an increase in specific heat capacity. The average specific heats of sample 2 in solid and molten states are 1.14 J/(g·K) and 1.91 J/(g·K), respectively, representing increases of 11.8% and 21.3% compared to the base salt. Sample 3 exhibits average specific heats of 1.15 J/(g·K) in the solid state and 2.36 J/(g·K) in the molten state, representing increases of 12.0% and 50.4%, respectively, compared to the base salt. The specific heats of the remaining samples are close to or lower than that of the base salt.

Understanding the mechanism by which nanoparticles affect the specific heat of molten salts is a prominent research focus in this field. Although at least three potential mechanisms have been suggested, agreement among scholars is still lacking [21,22,23,24,25]. The increase in the specific heat of the samples in this study may be attributed to several possible reasons. From an energy perspective, the phenomenon of strong interfacial molecular layers may be an important factor contributing to the enhancement of the specific heat. The interaction between nanoparticles and molten salt implies a correlation between van der Waals forces and Coulomb forces among particles, leading to the formation of an energy-storing force field, ultimately increasing the specific heat capacity [26]. In addition, the doping of nanoparticles in molten salt can influence grain size, enlarge interfacial area, and thereby contribute to a potential increase in specific heat capacity [9].

Most samples in this study contain two types of nanoparticles, exhibiting varying levels of specific heat capacities. There may be a synergistic effect between two types of nanoparticles and the base salt, which is linked to the concentration ratio of the nanoparticles [27]. The factors affecting synergistic effects are connected to the electrostatic forces between nanoparticles and ions. Mixing two distinct types of nanoparticles in varying proportions results in mutual charge interactions, thereby inducing modifications in the aggregation behavior of nanoparticles, and consequently influencing the specific heat of the composite material [8].

### 3.4. Heat Transfer Performance

**Figure 6:**
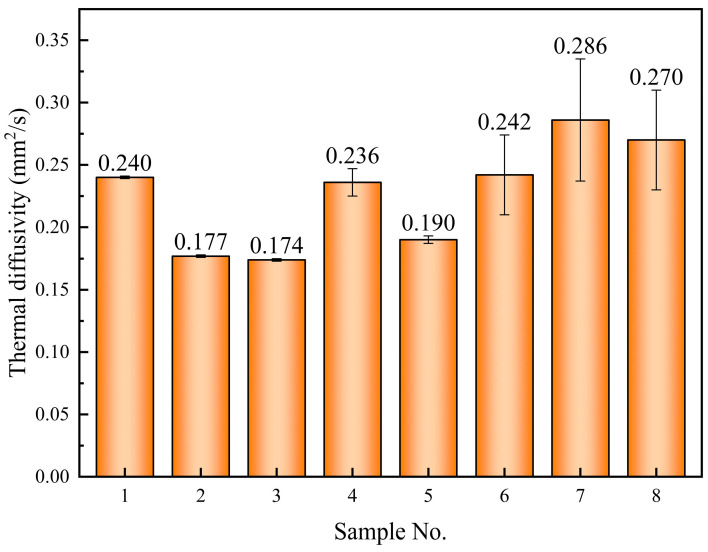
Thermal diffusivity coefficient of the samples in a solid state at 50 °C.

The thermal diffusivity of the sample in its solid state at 50 °C is shown in Figure 6. The thermal diffusion coefficient of the composite material reaches 0.270 mm^2^/s when 1% nano-SiO_2_ is added separately, showing a 12.5% increase compared to the base salt (0.240 mm^2^/s). Among all samples, sample 7 exhibited the highest thermal diffusivity at 0.286 mm^2^/s, showing an increase of 19.2%. It should also be noted that the thermal diffusivity of samples 2, 3, 4, and 5 has decreased, with reductions ranging from 1.7% to 27.5%.

**Figure 7:**
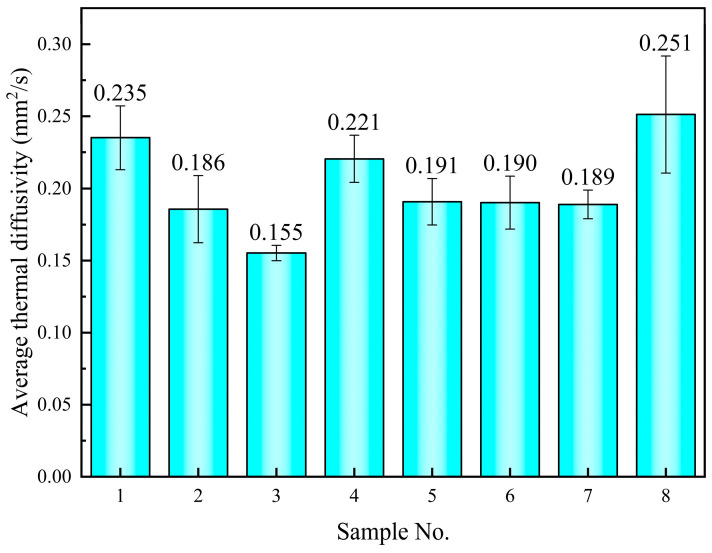
The average thermal diffusion coefficient of molten samples from 100 °C to 400 °C.

The thermal diffusivity coefficients of the samples in the molten state are shown in Figure 7 and Table 5. Upon the addition of 1% nano-SiO_2_, the thermal diffusion coefficient increases by 6.8% to 0.251 mm^2^/s from the base salt value of 0.235 mm^2^/s. A decrease in thermal diffusivity is observed in the rest of the samples, with reductions ranging from 6.0% to 34.0%. It is evident that the thermal diffusivity in the molten state is generally lower than in the solid state. This is attributed to the close-packed arrangement of the ions in solids, which enhances phonon heat transfer efficiency. In contrast, the disordered motion of ions in the molten state results in weaker inter-ionic bonds, impeding phonon propagation.

**Figure 8:**
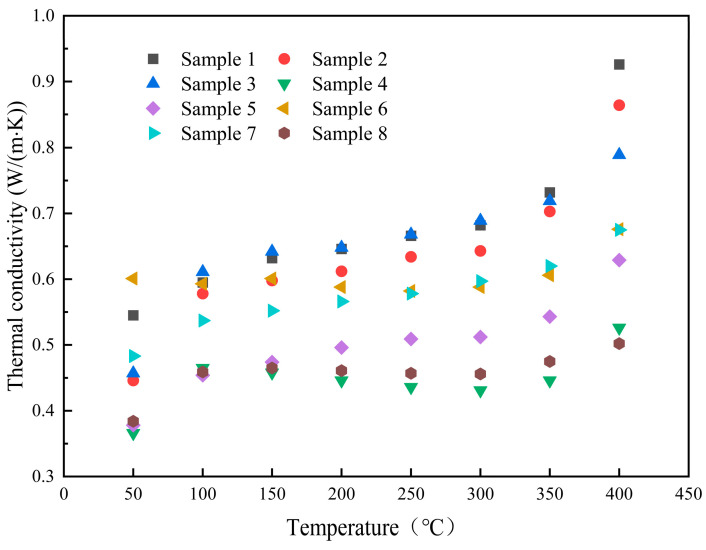
Thermal conductivity of the samples.

The thermal conductivity values of the samples in solid and molten states, along with their changes relative to the base salt, are presented in Table 6 and Table 7. Figure 8 distinctly shows the relative sizes of the thermal conductivity values. In the solid state, sample 6 demonstrates a higher thermal conductivity compared to the base salt, with an increase of 10.3% to 0.601 W/(m·K). The thermal conductivities of the other samples do not exhibit notable improvements in either solid or molten states. The factors that could impact the thermal conductivity of composite materials in this study may include the following aspects. Firstly, although the high density and strong interaction between cations and anions in the interfacial layer of composite materials are beneficial for enhancing thermal conductivity, the detrimental effect of interfacial thermal resistance is also significant. This negative impact outweighs the positive effect, leading to a decrease in thermal conductivity [28]. Additionally, the suboptimal dispersion and stability of nanoparticles in the base salt may lead to agglomeration, which, in turn, hinders interfacial contact and diminishes thermal energy transfer efficiency [29]. Finally, the thermal conductivity of nanoparticles is influenced by factors such as the particle size, density, specific surface area, and doping level. For instance, an excessive doping level can lead to increased agglomeration, consequently reducing the thermal conductivity [30,31]. Additionally, in light of recent research outcomes and the effective medium theory, the phenomenon of interfacial heat scattering could potentially contribute to the decrease in heat transfer efficiency in our low-doped system [32].

### 3.5. Microstructures

**Figure 9:**
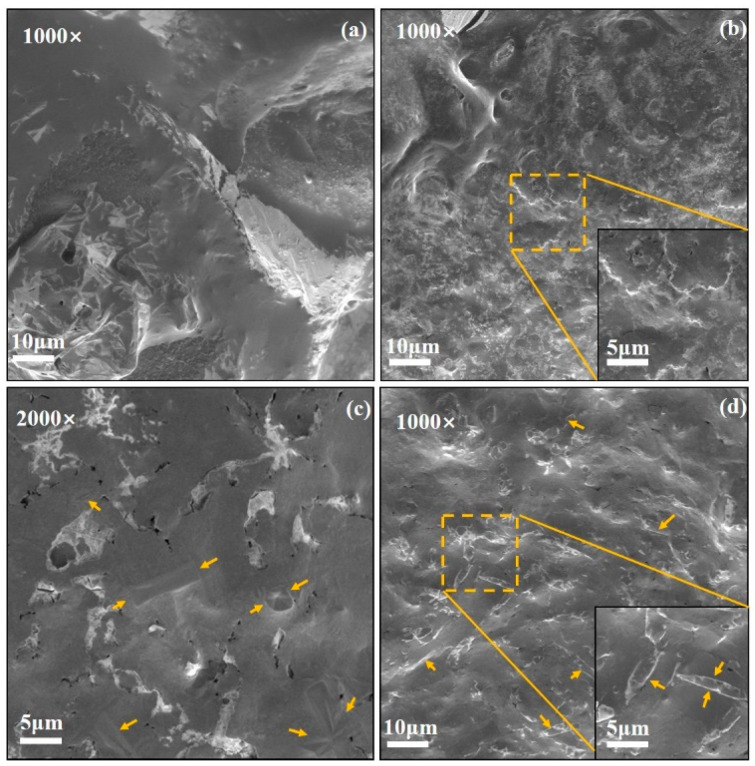
SEM image of (**a**) base salt, (**b**) 1%MgO-base salt, (**c**) 0.1%SiO_2_-0.9%MgO-base salt, and (**d**) 0.9%SiO_2_-0.1%MgO-base salt.

The results above indicate a notable increase in specific heat for samples 2 and 3, and a higher thermal diffusivity in the solid state of sample 7. The microstructures of these samples and the base salt are analyzed in this study, with the findings presented in Figure 9.

Figure 9b displays an image of sample 2, revealing numerous protrusions within the material, contrasting with the relatively flat surface of the base salt shown in Figure 9a. In Figure 9c, clear block-shaped crystals are observed on the surface of sample 3. The higher specific heat of samples 2 and 3 may be attributed to the significant interfacial thermal resistance introduced by their emerging microstructures. It is believed that there is an interfacial thermal resistance between the nanoparticle and the base salt, which results from their interaction. This interfacial thermal resistance can store extra thermal energy as a virtual spring-mass system [33].

The microstructure of sample 7, as depicted in Figure 9d, exhibits a notable presence of rod-like structures. This characteristic may be indicative of higher thermal diffusivity, as such structures exhibit enhanced phonon heat transfer efficiency compared to disordered structures.

## 4. Conclusions

In this study, molten salt nanocomposites were prepared through the microwave method using the quinary molten salt (LiNO_3_-NaNO_3_-KNO_3_-NaNO_2_-KNO_2_) and two types of nanoparticles (SiO_2_, MgO) as raw materials. The impacts of adding two types of nanoparticles at varying concentrations on the thermal and microstructural properties of composite materials were investigated. The main conclusions are as follows:

(1) The melting point of the base salt can be influenced by the doping of SiO_2_ and MgO nanoparticles, albeit with a limited fluctuation range (70–75 °C). The latent heat of the phase change in composite materials can also be affected by doping. Specifically, the doping of 0.5% SiO_2_ and 0.5% MgO nanoparticles resulted in a significant enhancement in latent heat, reaching 151.6 J/g, which represents a 6.2% increase compared to the base salt;
(2) The doping of 1 wt.% MgO or 0.1% SiO_2_-0.9% MgO leads to a significant increase in the specific heat capacity. The maximum specific heat capacity of the composite material is 2.36 J/(g·K), exhibiting a 50.4% enhancement over the base salt. Based on current research in the field, it is believed that the phenomenon may be attributed to the interfacial thermal resistance commonly found in composite materials or the electrostatic fields between nanoparticles;
(3) The doping of 0.9% SiO_2_-0.1% MgO significantly enhances the thermal diffusion coefficient of the base salt in its solid state, reaching 0.286 mm^2^/s, representing a 19.2% increase. The SEM images reveal the presence of numerous rod-like structures in the solid state of this sample, which can demonstrate favorable efficiency in phonon heat transfer;
(4) Although interface thermal resistance can enhance specific heat, it can also result in the reduction of heat transfer efficiency in materials. The specific heat capacities of samples 2 and 3 in this study are higher, while their thermal diffusivities are lower. This phenomenon is consistent with the characteristics of interfacial thermal resistance.

## Figures and Tables

**Table 1:** Properties of nitrate and nitrite.

Raw Material	Melting Point (°C)	Density (g/cm^3^)	Purity (%)
LiNO_3_	264	2.38	≥99.0
NaNO_3_	307	2.26	≥99.0
KNO_3_	334	2.11	≥99.0
NaNO_2_	271	2.17	≥99.0
KNO_2_	350 (decompose)	1.92	≥99.0

**Table 2:** Properties of nanoparticles.

Characteristics	Parameter	
Types of Nanoparticles	SiO_2_	MgO
Purity (%)	99.9	99
Average size (nm)	About 30	About 50
Specific surface area (m^2^/g)	2.65	3.58
Density (g/cm^3^)	143	50
Thermal conductivity (W/(m·K))	8.3	36
Specific heat (J/(g·K))	0.99	0.92

**Table 3:** The composition ratio of the samples.

Sample No.	LiNO_3_-NaNO_3_-KNO_3_-NaNO_2_-KNO_2_ (wt.%)	SiO_2_ Nanoparticles (wt.%)	MgO Nanoparticles (wt.%)
1	100	0	0
2	99	0	1.0
3	99	0.1	0.9
4	99	0.3	0.7
5	99	0.5	0.5
6	99	0.7	0.3
7	99	0.9	0.1
8	99	1.0	0

**Table 4:** Specific heat of the samples.

Sample No.	Specific Heat (J/(g·K))							
	50 °C	100 °C	150 °C	200 °C	250 °C	300 °C	350 °C	400 °C
1	1.05 (±0.10) *	1.54 (±0.03) *	1.51 (±0.07) *	1.52 (±0.08) *	1.54 (±0.09) *	1.56 (±0.11) *	1.65 (±0.14) *	1.77 (±0.19) *
2	1.16 (±0.04) *	1.92 (±0.02) *	1.90 (±0.03) *	1.88 (±0.04) *	1.92 (±0.04) *	1.86 (±0.09) *	1.88 (±0.13) *	2.01 (±0.16) *
3	1.21 (±0.21) *	2.13 (±0.03) *	2.27 (±0.02) *	2.30 (±0.06) *	2.37 (±0.08) *	2.39 (±0.11) *	2.44 (±0.14) *	2.55 (±0.14) *
4	0.72 (±0.31) *	1.31 (±0.10) *	1.17 (±0.10) *	1.11 (±0.12) *	1.02 (±0.15) *	0.98 (±0.23) *	1.03 (±0.19) *	1.23 (±0.18) *
5	0.92 (±0.20) *	1.42 (±0.05) *	1.44 (±0.04) *	1.45 (±0.01) *	1.45 (±0.04) *	1.42 (±0.06) *	1.43 (±0.06) *	1.55 (±0.09) *
6	1.15 (±0.28) *	2.00 (±0.04) *	1.87 (±0.06) *	1.69 (±0.13) *	1.59 (±0.19) *	1.55 (±0.24) *	1.57 (±0.27) *	1.77 (±0.37) *
7	0.78 (±0.15) *	1.68 (±0.11) *	1.61 (±0.10) *	1.58 (±0.08) *	1.57 (±0.06) *	1.60 (±0.04) *	1.71 (±0.02) *	1.99 (±0.17) *
8	0.66 (±0.20) *	1.30 (±0.02) *	1.18 (±0.01) *	1.05 (±0.02) *	0.94 (±0.03) *	0.89 (±0.04) *	0.88 (±0.05) *	0.91 (±0.05) *

* Standard deviation of data obtained from three specific heat tests.

**Table 5:** The average thermal diffusion coefficient (mm^2^/s) and the change rate of the samples.

Sample No.	100 °C	200 °C	300 °C	400 °C	Average Value	Change Rate
1	0.208 (±0.008) *	0.228 (±0.001) *	0.235 (±0.000) *	0.280 (±0.001) *	0.235	-
2	0.162 (±0.004) *	0.174 (±0.001) *	0.185 (±0.000) *	0.231 (±0.001) *	0.186	−20.9%
3	0.154 (±0.001) *	0.151 (±0.000) *	0.155 (±0.000) *	0.166 (±0.002) *	0.155	−34.0%
4	0.190 (±0.003) *	0.217 (±0.000) *	0.236 (±0.001) *	0.229 (±0.003) *	0.221	−6.0%
5	0.171 (±0.003) *	0.184 (±0.001) *	0.194 (±0.000) *	0.218 (±0.001) *	0.191	−18.7%
6	0.159 (±0.002) *	0.187 (±0.000) *	0.203 (±0.000) *	0.205 (±0.003) *	0.190	−19.1%
7	0.172 (±0.002) *	0.192 (±0.000) *	0.200 (±0.001) *	0.182 (±0.003) *	0.189	−19.6%
8	0.189 (±0.004) *	0.236 (±0.000) *	0.274 (±0.000) *	0.297 (±0.004) *	0.251	6.8%

* Standard deviation of data obtained from three specific heat tests.

**Table 6:** Thermal conductivity (W/(m·K)) and the change rate of the samples at 50 °C in solid stat.

Sample No.	Thermal Conductivity	Change Rate
1	0.545 (±0.001) *	-
2	0.446 (±0.002) *	−18.2
3	0.457 (±0.001) *	−16.1%
4	0.366 (±0.009) *	−32.8%
5	0.378 (±0.004) *	−30.6%
6	0.601 (±0.040) *	10.3%
7	0.483 (±0.042) *	−11.4%
8	0.384 (±0.029) *	−29.5%

* Standard deviation of data obtained from three specific heat tests.

**Table 7:** Thermal conductivity (W/(m·K)) and the change rate of the samples in the molten state.

Sample No.	100 °C	200 °C	300 °C	400 °C	Average Value	Change Rate
1	0.595 (±0.022) *	0.646 (±0.001) *	0.682 (±0.000) *	0.926 (±0.003) *	0.697	-
2	0.578 (±0.012) *	0.612 (±0.001) *	0.643 (±0.001) *	0.864 (±0.003) *	0.662	−5.0%
3	0.611 (±0.002) *	0.648 (±0.001) *	0.689 (±0.001) *	0.789 (±0.007) *	0.681	−2.3%
4	0.465 (±0.008) *	0.446 (±0.001) *	0.431 (±0.003) *	0.526 (±0.007) *	0.458	−34.3%
5	0.454 (±0.007) *	0.496 (±0.001) *	0.512 (±0.001) *	0.629 (±0.001) *	0.517	−25.8%
6	0.593 (±0.009) *	0.588 (±0.001) *	0.588 (±0.001) *	0.676 (±0.009) *	0.605	−13.2%
7	0.537 (±0.005) *	0.566 (±0.001) *	0.597 (±0.001) *	0.675 (±0.011) *	0.589	−15.5%
8	0.459 (±0.010) *	0.461 (±0.001) *	0.456 (±0.001) *	0.502 (±0.006) *	0.468	−32.9%

* Standard deviation of data obtained from three specific heat tests.

## Data Availability

The original contributions presented in this study are included in the article; further inquiries can be directed to the corresponding author.
